# Supplementary material for: The causal relationship between the human gut microbiota and pyogenic arthritis: a Mendelian randomization study
Source: Front Cell Infect Microbiol. 2024 Nov 26;14:1452480. doi: 10.3389/fcimb.2024.1452480 (PMC11629706; doi:10.3389/fcimb.2024.1452480)
Supplement: Supplementary file 1 [file DataSheet1.zip › mendelian test/ebi-a-GCST90027645.csv_forest.pdf]

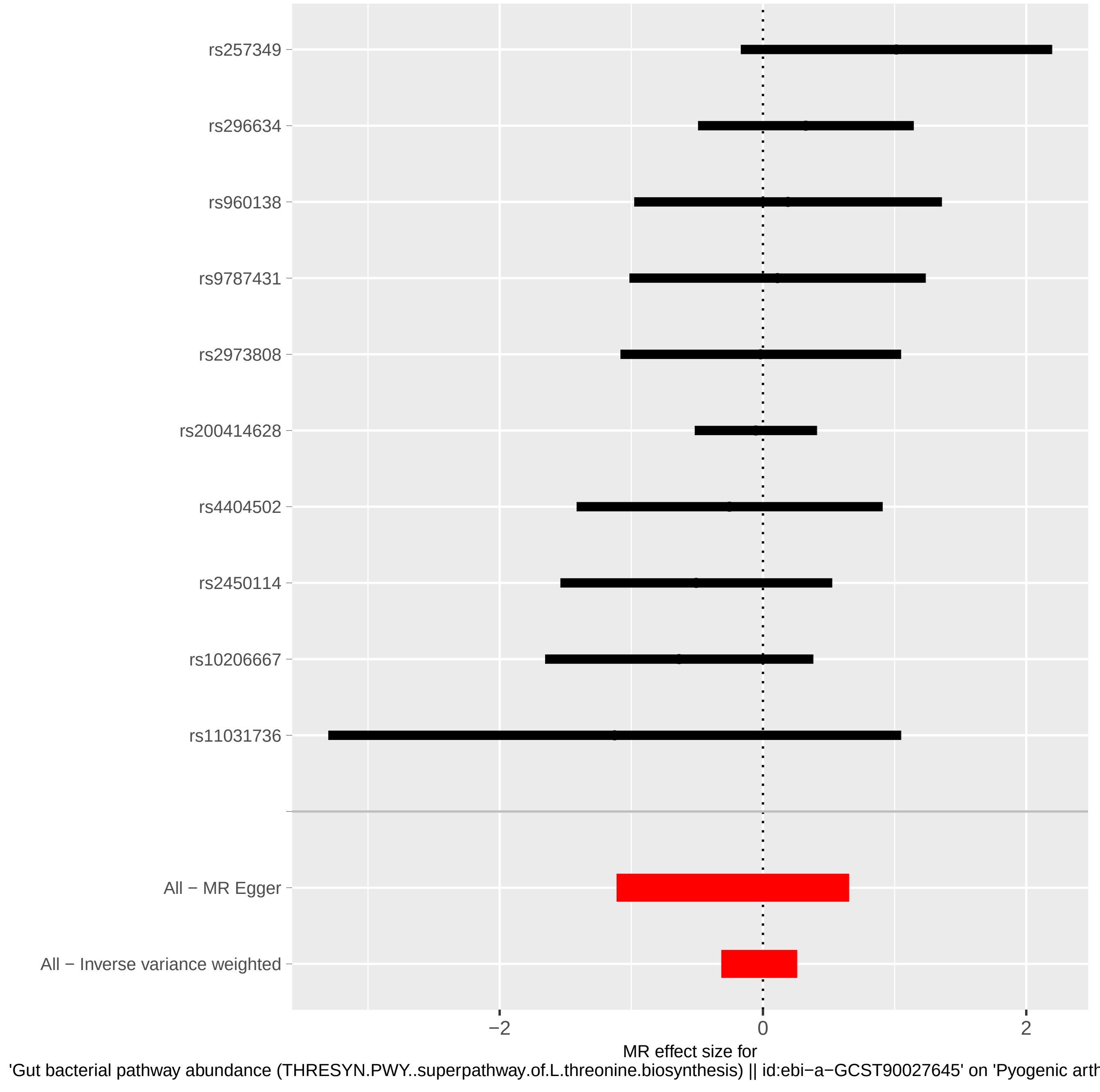
rs257349
rs296634
rs960138
rs9787431
rs2973808
rs200414628
rs4404502
rs2450114
rs10206667
rs11031736
All − MR Egger
All − Inverse variance weighted
−2
0
2
MR effect size for
'Gut bacterial pathway abundance (THRESYN.PWY..superpathway.of.L.threonine.biosynthesis) || id:ebi−a−GCST90027645' on 'Pyogenic arth
